# Correction: *In vivo* study of the radioadaptive response and low-dose hyper-radiosensitivity for chromosome breaks induced by gamma rays in wild-type *Drosophila melanogaster* larval neuroblasts: Dose and dose rate dependence

**DOI:** 10.1371/journal.pone.0332063

**Published:** 2025-09-10

**Authors:** Claudia Di Dio, Antonella Porrazzo, Alex De Gregorio, Patrizia Morciano, Maria Antonella Tabocchini, Giovanni Cenci, Francesca Cipressa, Giuseppe Esposito

The corresponding author’s email address is incorrect. Francesca Cipressa’s email address is: francesca.cipressa@unitus.it.
