# Supplementary figures and images for: In vivo study of the radioadaptive response and low-dose hyper-radiosensitivity for chromosome breaks induced by gamma rays in wild-type Drosophila melanogaster larval neuroblasts: Dose and dose rate dependence
Source: PLoS One. 2025 Jun 9;20(6):e0325608. doi: 10.1371/journal.pone.0325608 (PMC12148174; doi:10.1371/journal.pone.0325608)

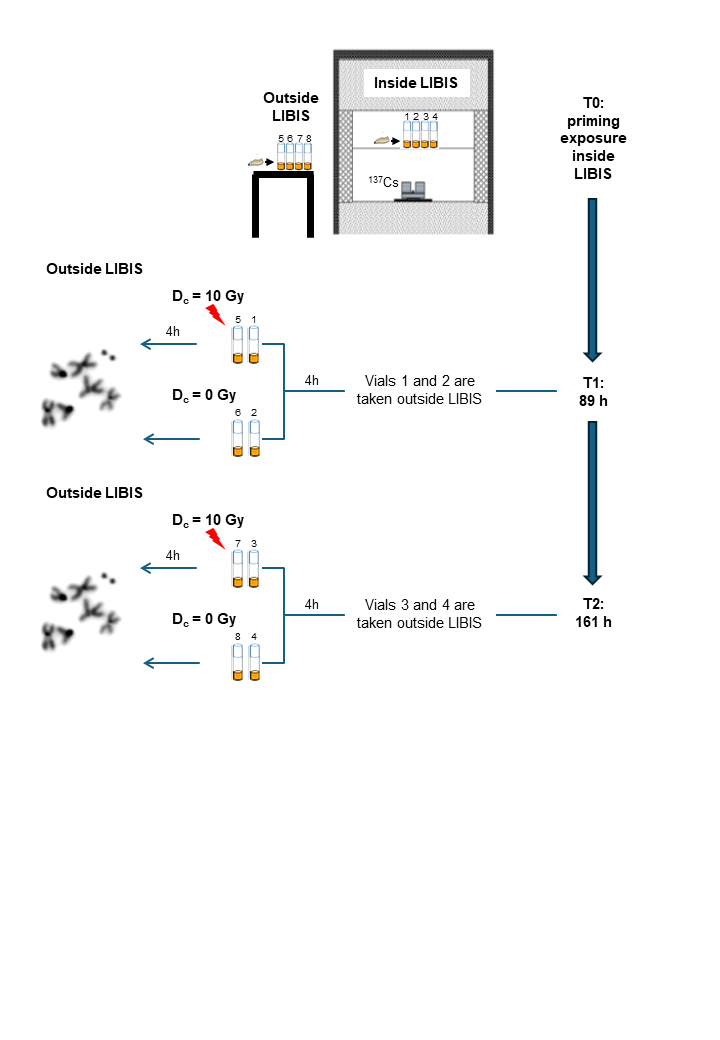

Supplement: S1 Fig — Vials containing Drosophila embryos were placed in the LIBIS irradiator at different distances from the 137Cs source and exposed to continuous irradiation at different dose rates until they developed into third instar larvae. 89 and 161 hours after the start of the priming exposure, vials (containing third instar larvae) were taken out of LIBIS and some of them were irradiated with an additional 10 Gy of γ-rays (challenging dose). At both the time points, even unpretreated vials containing third instar larvae were exposed to the challenging dose alone. Moreover, vials that were not exposed to either the priming dose or the challenging dose were also considered in our study. Subsequently, all vials were analysed to determine the frequency of CBs. (TIF) [file pone.0325608.s001.tif]
